# Subspace Identification of Bridge Frequencies Based on the Dimensionless Response of a Two-Axle Vehicle

**DOI:** 10.3390/s24061946

**Published:** 2024-03-18

**Authors:** Yixin Quan, Qing Zeng, Nan Jin, Yipeng Zhu, Chengyin Liu

**Affiliations:** 1 School of Civil and Environmental Engineering, Harbin Institute of Technology (Shenzhen), Shenzhen 518055, China; 21s154140@stu.hit.edu.cn (Y.Q.); zengqing@hit.edu.cn (Q.Z.); 22b954015@stu.hit.edu.cn (Y.Z.); 2 Guangdong Provincial Key Laboratory of Intelligent and Resilient Structures for Civil Engineering, Shenzhen 518055, China; 3 Key Laboratory of Urban Safety Risk Monitoring and Early Warning, Ministry of Emergency Management, Shenzhen 518055, China; jinnan@szsti.org; 4 Shenzhen Technology Institute of Urban Public Safety, Shenzhen 518023, China

**Keywords:** bridge frequency identification, vehicle scanning method, subspace identification method, vehicle–bridge interaction analysis, dimensionless parameter analysis

## Abstract

As an essential reference to bridge dynamic characteristics, the identification of bridge frequencies has far-reaching consequences for the health monitoring and damage evaluation of bridges. This study proposes a uniform scheme to identify bridge frequencies with two different subspace-based methodologies, i.e., an improved Short-Time Stochastic Subspace Identification (ST-SSI) method and an improved Multivariable Output Error State Space (MOESP) method, by simply adjusting the signal inputs. One of the key features of the proposed scheme is the dimensionless description of the vehicle–bridge interaction system and the employment of the dimensionless response of a two-axle vehicle as the state input, which enhances the robustness of the vehicle properties and speed. Additionally, it establishes the equation of the vehicle biaxial response difference considering the time shift between the front and the rear wheels, theoretically eliminating the road roughness information in the state equation and output signal effectively. The numerical examples discuss the effects of vehicle speeds, road roughness conditions, and ongoing traffic on the bridge identification. According to the dimensionless speed parameter *S_v_*_1_ of the vehicle, the ST-SSI (*S_v_*_1_ < 0.1) or MOESP (*S_v_*_1_ ≥ 0.1) algorithm is applied to extract the frequencies of a simply supported bridge from the dimensionless response of a two-axle vehicle on a single passage. In addition, the proposed methodology is applied to two types of long-span complex bridges. The results show that the proposed approaches exhibit good performance in identifying multi-order frequencies of the bridges, even considering high vehicle speeds, high levels of road surface roughness, and random traffic flows.

## 1. Introduction

As one of the most essential components of the transportation system and the urban transportation hub, municipal bridges help solve the problem of traffic clogs and facilitate the optimization of the traffic flow, a situation commonly found in most areas of the world [1]. Since the reform and opening up policy, China has seen quantum leaps in this regard over the past decades, with its volume of bridges in use surpassing the one million mark by the end of 2022. However, a vast number of existing bridges following outdated design standards have served a long time over an extended period. Various natural disasters and environmental disturbances have deteriorated the health of bridge structures, underscoring the pressing need for a fast and effective monitoring method to sustain the operational safety of bridges.

As a crucial reference of structural characteristics, bridge modal parameters provide comprehensive information for healthy bridge structures and reflect the damage to some extent [2,3,4,5]. Consequently, acquiring structural modal parameters has become a global focus of attention. Conventional methods, also known as *direct methods*, involve numerous sensors installed along the bridge to monitor the vibration response excited by traversing vehicles or other excitations. Over the past decades, scholars have carried out a large amount of research on these direct methods [6,7,8,9]. The limitation of the *direct methods* lies in the high cost of equipment installation and maintenance, together with the manual labor consumption and the interruption of the traffic flow [10].

To overcome this challenge, most recently, researchers have proposed a series of *indirect methods* to identify the bridge modal parameters. The most feasible and applicable method is to extract the bridge’s modal parameters from the vehicle dynamic response, known as the vehicle scanning method (VSM). Later, the VSM developed into mainly two categories. One is based on spectral analysis from the vehicle response, and the other is the subspace identification method. Yang et al. [11] theoretically proposed the concept of treating a passing vehicle as a loading exciter as well as a *message carrier,* collecting dynamic information about the bridge indirectly through the traversing vehicle. Several field tests have demonstrated the feasibility and reliability of using the VSM to extract bridge frequencies indirectly [12,13]. In recent years, the VSM has gained popularity for identifying frequencies of bridge systems [14,15,16,17,18]. Many scholars all around the world have carried out research based on the VSM, and an array of advanced filtering techniques have been used to extract the bridge frequencies from the vehicle signals, such as the empirical modal decomposition (EMD) [19], the variational modal decomposition (VMD) [20], and the stochastic subspace (SSI) methods [21]. Later, Yang et al. [22] proposed a combination of band-pass filtering and singular value spectral analysis (SSA-BPF) to deal with vehicle signals, suggesting that the proposed method can effectively filter out the vehicle frequencies to facilitate the identification of the bridge frequencies. Nagayama et al. [23] introduced a cross-spectral density function to extract the common component in the responses of two moving vehicles, which is assumed to be representative of the bridge vibration as well. They validated its practical effectiveness by employing a field test. Recently, Liu et al. [24] transferred the tire pressure monitoring signals to the axle-contact point response of a vehicle passing over a bridge to extract the frequency of the bridge. The vehicle transverses the bridge twice to calculate the residual bridge displacement and to compensate for the effect of pavement roughness as well. Compared with *direct methods*, the VSM is economically attractive and enables a quick and efficient diagnosis of urban bridge systems on a large scale without disrupting normal traffic operations. Despite all these merits, the VSM encounters specific challenges in its applications, including adverse effects of the road surface roughness [25,26], difficulties in extracting higher-order frequencies of the bridge [27,28,29], and the possibility of the vehicle frequencies exceeding those of the bridge [22,30,31] and driving speed limitations [14,32,33].

In view of these cruxes, Yang et al. [31] theoretically validated that the contact point response is unaffected by the vehicle’s frequency and is superior to the vehicle response in identifying bridge frequencies. Later, Xu et al. [34] explored the effect of vehicle damping on the contact point response. Further, Xu et al. [35] adopted a two degrees of freedom (DOFs) vehicle system, taking into account the suspension effect, and investigated the responses of the vehicle body, wheel, and contact point in identifying the bridge frequencies. In general, the contact point response has been shown to be more capable of retrieving the first few orders of frequencies of the bridge than other responses. However, for multi-axle vehicles in which it is more difficult to install the sensors and measure the contact point response, Yang et al. [36] turned to more feasible means by making the vehicle’s frequency much higher than that of the bridge; thus, the concept of a frequency-free measurement vehicle was proposed. To better eliminate the adverse effects of the road roughness and vehicle frequency simultaneously, Yang et al. [37] intensified the bridge vibration by introducing a shaker. It was found that the shaker facilitates the VSM for bridges with rough surfaces, and that the combination of the contact response and a shaker has a wider range of applications. Meanwhile, a scheme considering both a static vehicle and a moving one has also been proposed and is proven to be more advantageous in increasing the extractability of bridge frequencies [30,38,39].

In 2015, Yang et al. [21] proposed the stochastic subspace method (SSI) to process the response of the *single-degree-of-freedom* (SDOF) vehicle. They successfully separated the bridge information from the vehicle response through a series of algorithms for the equation of motion (EOM) of the coupled vehicle–bridge interaction (VBI) system, indirectly identifying the frequencies of a simply supported bridge. Subsequently, Jin et al. [40] proved the SSI method by employing a *multi-degree-of-freedom* (MDOF) vehicle and a finite element bridge model, finding the ongoing traffic flow effectively induced high-order modal vibrations in the bridge, facilitating the extraction of high-order bridge frequency from the vehicle signal. Since then, the SSI has been further improved and updated. Li et al. [41] successfully obtained both the bridge frequencies and mode shapes using SSI through two instrumented vehicles—one serving as a fixed reference point and another as a moving sensor. Eshkevari et al. [17] combined the second-order blind identification method and extended structural modal identification using expectation maximization for modal identification of complicated bridges using sensor data collected from moving vehicles. More recently, Jin et al. [42] proposed a Short-Time Stochastic Subspace Identification (ST-SSI) method, incorporating the concept of a dimensionless velocity parameter to address the indirect identification of bridge frequencies using traversing vehicles at high speed. Afterward, they adopted another subspace method [43], the Multivariable Output Error State Space (MOESP), approximating the first-order mode shape of the bridge and utilizing the pseudo-inverse matrix algorithm based on singular value decomposition to break the limitation on the traversing vehicle speed for the indirect method. Both the ST-SSI and MOESP approaches overcame the limitations associated with the time-varying characteristics of the vehicle–bridge coupling system, making it advantageous for frequency identification.

In most cases, one can employ either ST-SSI or the MOESP [42,43] to identify different types of bridges, disregarding the natural time-variation of the VBI system. However, the previous studies [42,43] request the vehicle to cross the bridge twice with a precise and fixed speed for the purpose of removing the road roughness conditions (RRCs), which is theoretically applicable but practically infeasible. To overcome the aforementioned limitations, the study extends ST-SSI and the MOESP and proposes an improved subspace-based identification method considering a *time shift* between two wheels of a two-axle vehicle., allowing the elimination of the adverse effect of the RRCs from the dimensionless response of a passing vehicle on a single drive. More importantly, a dimensionless description of the vehicle–bridge interaction system (not rarely the dimensionless speed) and the employment of the dimensionless response of a two-axle vehicle as the state input enhances the robustness to the vehicle properties and speed. Additionally, a procedure for data processing completely removes the negative noise for the identification process, so the scheme does not rely on vehicle properties. Through comprehensive numerical examples, the study validates the feasibility of subspace identification methods (i.e., ST-SSI and MOESP) for multi-order frequency identification of bridges with complex structural forms to provide theoretical support for practical engineering applications. At the same time, the influence of driving speed, level of road roughness, and random traffic flow in practical applications on the method is discussed. In addition, the subspace identification method is more suitable for ordinary vehicles and driving environments, which provides further theoretical support for the practical engineering application of the method.

## 2. Theoretical Formulation

### 2.1. A Brief Review of Subspace Identification

Conventional subspace identification methods identify the state space model of the system by directly processing the input and output data in the time domain. The response of a bridge structure subjected to dynamic loads under ambient excitations is described by a discrete stochastic state space model as

(1) $$X_{k+1}=AX_{k}+BU_{k}+w_{k}$$

(2) $$Y_{k}=CX_{k}+DU_{k}+v_{k}$$

where **X***_k_* represents the state vector; **A**, **B**, **C,** and **D** denote the time-invariant system matrices; **w***_k_* and **v***_k_* are system noise and measurement noise, respectively, which are treated as zero-mean white noise. The main purpose of the subspace identification method (i.e., MOESP) is to determine the system matrix (**A**, **B**, **C** and **D**) through the input and output signals (i.e., **U***_k_* and **Y***_k_*), given later on.

In particular, ST-SSI is used for the output-only dynamic system (Equations (3) and (4)), with the input signals **U***_k_* removed.

(3) $$X_{k+1}=AX_{k}+w_{k}$$

(4) $$Y_{k}=CX_{k}+v_{k}$$

Contrary to conventional SSI, both ST-SSI and the MOESP are applicable to time-varying systems [43]. The point is that ST-SSI and the MOESP allow the time variation of system matrices **A** and **C** in particular scenarios when the time variation is trivial compared to the system vibration. To this end, a dimensionless speed parameter $S_{vn}=\omega_{Ln}/\omega_{n}^{B}$ is introduced as an indicator of the variation. Defined by the ratio of the loading frequency $\omega_{Ln}$ to the bridge frequency $\omega_{n}^{B}$, the dimensionless speed reflects the changing rate of the dynamic load.

(5) $$\omega_{Ln}=\frac{n\pi v}{L_{eff}}$$

where *L_eff_* represents the characteristic length of a bridge. The dimensionless speed parameter *S_vn_* can be written as *S_v_*_1_ regarding solely the first mode (*n* = 1) of the bridge.

Meanwhile, a value of 0.1 for *S_v_*_1_ (*n* = 1) is set to limit the scope of application of ST-SSI [42]. The dynamic characteristics of the bridge can be considered negligible relative to that of the vehicle when the dimensionless speed is less than 0.1. Therefore, the VBI problem can be referred to as a “static” problem.

When the dimensionless speed exceeds this value 0.1, the time variation of the system is non-negligible. With the aid of the inverse matrix algorithm, the MOESP removes the time-varying components in the system matrix **C**. Therefore, there is no speed limitations for the MOESP [43].

### 2.2. Formulation of VBI System with Dimensionless Terms

**Figure 1:**
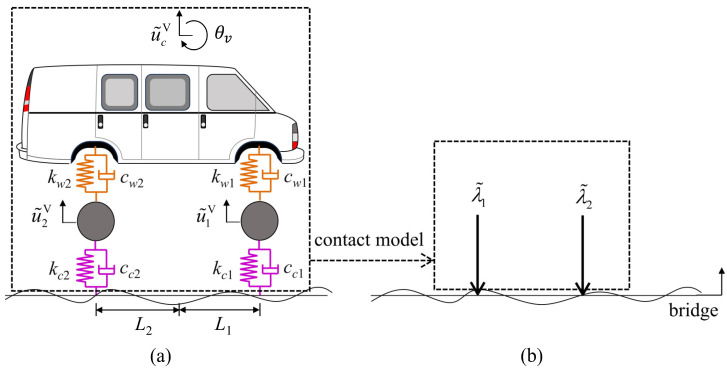
A two-axle MDOF vehicle model: (**a**) vehicle model (**b**) simplified contact force.

In the process of a MDOF vehicle (Figure 1a) driving across a (generic) bridge at a constant speed *v*. The equations of motion (EOM) for the bridge system can be given as

(6) $$M^{B}{\ddot{\tilde{u}}}^{B}\left(t\right)+C^{B}{\dot{\tilde{u}}}^{B}\left(t\right)+K^{B}{\tilde{u}}^{B}\left(t\right)+W_{N}^{B}\left(\tilde{x}\right)\tilde{\lambda }\left(t\right)={\tilde{F}}_{G}$$

and, simultaneously, the EOM for the vehicle system as

(7) $$M^{V}{\ddot{\tilde{u}}}^{V}\left(t\right)+C^{V}{\dot{\tilde{u}}}^{V}\left(t\right)+K^{V}{\tilde{u}}^{V}\left(t\right)-W_{N}^{V}\left(\tilde{x}\right)\tilde{\lambda }\left(t\right)=0$$

where $M^{B/C}$, $C^{B/C}$, and $K^{B/C}$ are the mass, damping, and stiffness matrices of the bridge/vehicle, respectively. ${\tilde{u}}^{B}(t)$ and ${\tilde{u}}^{V}(t)$ are the displacement vectors of the bridge and vehicle, respectively, with ${\tilde{u}}^{V}\left(t\right)={\left[\begin{array}{cccc} {\tilde{u}}_{c}^{V}\left(t\right) & {\tilde{\theta }}_{v}\left(t\right) & {\tilde{u}}_{1}^{V}\left(t\right) & {\tilde{u}}_{2}^{V}\left(t\right) \end{array}\right]}^{T}$. ${\tilde{F}}_{G}$ is the external force taking into consideration the weight of the vehicle. $W_{N}^{V}(\tilde{x})$ and $W_{N}^{B}(\tilde{x})$ are the direction matrices connecting the position and the direction of the contact forces $\tilde{\lambda }\left(t\right)$ with the DOFs of the bridge and the wheels, respectively. $\tilde{x}=vt$ represents the position of the wheel. $W_{N}^{B}(\tilde{x})$ is composed of the equation of shape function [42], and $W_{N}^{V}(\tilde{x})$ is of a matrix of 0 and 1:

(8) $$W_{N}^{V}\left(\tilde{x}\right)={\left[\begin{array}{cccc} 0 & 0 & 1 & 0\\ 0 & 0 & 0 & 1 \end{array}\right]}^{T}$$

With the help of the shape function of the bridge element [42], the contact force can be derived as

(9) $$\begin{array}{l} {\tilde{\lambda }}_{j}(t)=k_{cj}(-{\tilde{u}}_{j}^{V}(t)+W_{N}^{B}({\tilde{x}}_{j}{)}^{T}{\tilde{u}}^{B}(t)+r_{c}({\tilde{x}}_{j}))\\ +c_{cj}(-\frac{d}{dt}{\tilde{u}}_{j}^{V}(t)+W_{N}^{B}({\tilde{x}}_{j}{)}^{T}\frac{d}{dt}{\tilde{u}}^{B}(t)+v\frac{d}{d{\tilde{x}}_{j}}W_{N}^{B}({\tilde{x}}_{j}{)}^{T}{\tilde{u}}^{B}(t)+v\frac{d}{d{\tilde{x}}_{j}}r_{c}({\tilde{x}}_{j})) \end{array}$$

where the subscript *j* = 1, 2 represents the front/rear wheels of the vehicle. $k_{cj}$ and $c_{cj}$ are, respectively, the stiffness and damping of the tire. $r_{c}\left({\tilde{x}}_{j}\right)$ represents the height of the road roughness at the position of contact force.

For the sake of the proposed subspace identification, the interaction relationship between the vehicle and bridge is cast into the form of Equation (4) for the EOM of the contact force (Equation (9)) and Equation (3) for the EOM of the bridge (Equation (6)). In addition, due to the large number of parameters contained in Equations (6) and (9), then multiplying Equation (9) by ${1/m_{1}^{V}(\omega_{1}^{V})}^{2}L^{B}$ and using the dimensionless time *τ*, the contact force equations are now re-expressed as

(10) $$\begin{array}{ll} \lambda_{j}+K_{j}^{V}u_{j}^{V}+2\frac{C_{j}^{V}}{\omega_{1}^{V}}{\dot{u}}_{j}^{V} & =\left(K_{j}^{V}W_{N}^{B}{\left(x_{j}\right)}^{T}+\frac{2vC_{j}^{V}}{\omega_{1}^{V}L^{B}}W_{N}^{B}\left(x_{j}\right)\prime^{T}\right)u^{B}\\ & +2\frac{C_{j}^{V}}{\omega_{1}^{V}}W_{N}^{B}{\left(x_{j}\right)}^{T}{\dot{u}}^{B}+K_{j}^{V}R\left(x_{j}\right)+\frac{2vC_{j}^{V}}{\omega_{1}^{V}L^{B}}R{\left(x_{j}\right)}^{\prime } \end{array}$$

where *L*^B^ is the effective bridge length, with the full length for a simply supported beam bridge. $m_{1}^{V}$ and $\omega_{1}^{V}$ represent the first-order modal mass and frequency of the vehicle, respectively; $u_{j}^{V}={\tilde{u}}_{j}^{V}\left(t\right)/L^{B}$ represents the dimensionless displacement response of the *j*-th wheel of the vehicle on the displacement level; $u^{B}={\tilde{u}}^{B}\left(t\right)/L^{B}$ is the dimensionless bridge response on the displacement level; ${\dot{u}}_{j}^{V}$ and ${\dot{u}}^{B}$ represent the dimensionless response of the vehicle and bridge on the velocity level, respectively (derivative of the displacement response with respect to time *t*). $K_{j}^{V}=k_{cj}/m_{1}^{V}{\left(\omega_{1}^{V}\right)}^{2}$ and $C_{j}^{V}=c_{cj}/2m_{1}^{V}\omega_{1}^{V}$ denote the stiffness ratio and damping ratio of the *j*-th wheel of the vehicle, respectively; $x_{j}={\tilde{x}}_{j}/L^{B}$ denotes the dimensionless position; $R(x_{j})=r_{c}\left(x_{j}\right)/L^{B}$ denotes the scaled road roughness profile. ${\left(\right)}^{\prime }$ represents the derivative with respect to the dimensionless position parameter $x_{j}$.

### 2.3. Elimination of Adverse Impact of Road Roughness

It is worth noting that during the traverse, the front and rear wheels of the vehicle pass through the same route, thus the road roughness term of the two wheels is the same, with a distance of $l_{w}$:$R\left(x_{1}\right)=R\left(x_{2}+l_{w}/L^{B}\right)$. Similarly, the position matrix holds that $W_{N}^{B}\left(x_{1}\right)=W_{N}^{B}\left(x_{2}+l_{w}/L^{B}\right)$.

The contact stiffness $k_{cj}$ and damping coefficient $c_{cj}$ of the vehicle can be assumed as identical: $K_{1}^{V}\approx K_{2}^{V}\approx K^{V}$ and $C_{1}^{V}\approx C_{2}^{V}\approx C^{V}$. Then, dividing Equation (10) by $K^{V}$ and then subtracting the two equations yields the equation of residual dimensionless contact forces of two wheels:

(11) $$\begin{array}{l} \frac{1}{K^{V}}\Delta \lambda +\Delta u^{V}+\frac{2C^{V}}{\omega_{1}^{V}K^{V}}\Delta {\dot{u}}^{V}\\ \;\;\;\;\;\;\;\;\;\;=\left(W_{N}^{B}{\left(x_{j}\right)}^{T}+\frac{2vC^{V}}{\omega_{1}^{V}L^{B}K^{V}}W_{N}^{B}\left(x_{j}\right)\prime^{T}\right)\Delta u^{B}+\frac{2C^{V}}{\omega_{1}^{V}K^{V}}W_{N}^{B}{\left(x_{j}\right)}^{T}\Delta {\dot{u}}^{B} \end{array}$$

where $\Delta u^{V}=u_{1}^{V}(t)-u_{2}^{V}(t+t_{w})$, $\Delta u^{B}=u_{1}^{B}(t)-u_{2}^{B}(t+t_{w})$, and ∆*λ = λ*_1_(*t*) *− λ*_2_(*t + t_w_*) represent the residual dimensionless vehicle and bridge displacements, as well as the residual dimensionless contact force, respectively, considering the *time shift*.

Note that the term RRC is removed after the subtraction of the two wheels. Therefore, there is no restriction on choosing vehicle parameters in comparison with previous studies [42,43].

Accordingly, the EOM of the (dimensionless) residual response $\Delta u^{B}$ of the bridge is re-expressed as

(12) $$M^{B}\Delta {\ddot{u}}^{B}+C^{B}\Delta {\dot{u}}^{B}+K^{B}\Delta u^{B}=-\frac{1}{L^{B}}W_{N}^{B}(x)\Delta \tilde{\lambda }$$

### 2.4. Formulation of VBI Problem for System Identification

For the purpose of casting Equations (11) and (12) into the applicable state space form (Equations (3) and (4)) for the ST-SSI, the EOM of the contact force Equation (11) can be re-written in a state space form as

(13) $$\Delta P=N^{Vf}N^{WB}\left(x\right)\Delta X^{B}$$

where ∆*P* represents the output signal of the system and is made up of the vehicle parameters and response.

(14) $$\Delta P=\frac{1}{K^{V}}\Delta \lambda +\Delta u^{V}+\frac{2C^{V}}{\omega_{1}^{V}K^{V}}\Delta {\dot{u}}^{V}$$

It consists of the displacements and velocities at the front and rear wheel axles, as well as dynamic contact forces of both front and rear wheels. Practically, these displacements and velocities can be obtained indirectly by means of integrating the vertical accelerations measured by accelerators mounted on both axles. The contact forces are calculated by multiplying the relative contact displacement (that between the wheel axle and bridge provided by a laser) and the tire stiffness based on the elastic contact model [24]. Alternatively, the contact force can be reflected by the dynamic tire pressure [44]. $N^{Vf}$ and $N^{WB}(x)$ are the system matrices of the vehicle, defined as

(15) $$N^{Vf}=\left[\begin{array}{cc} 1 & \frac{2C^{V}}{\omega_{1}^{V}K^{V}} \end{array}\right],N^{WB}\left(x\right)=\left[\begin{array}{cc} W_{N}^{B}{\left(x\right)}^{T} & 0\\ \frac{v}{L^{B}}W_{N}^{B}\left(x\right)\prime^{T} & W_{N}^{B}{\left(x\right)}^{T} \end{array}\right]$$

respectively. Then the equation of output signal (Equation (13)) in state space takes the time-discrete form of

(16) $$\Delta P_{k}=N^{Vf}N_{k}^{WB}\Delta X_{k}^{B}$$

where $\Delta P_{k}$ is the known output signal for the ST-SSI method related to the vehicle at time step *k*,

(17) $$\Delta P_{k}=\frac{1}{K^{V}}\Delta \lambda_{k}+\Delta u_{k}^{V}+\frac{2C^{V}}{\omega_{1}^{V}K^{V}}\Delta {\dot{u}}_{k}^{V}$$

and $\Delta X_{k}^{B}=\left[\Delta u_{k}^{B}\right.{\left.\Delta {\dot{u}}_{k}^{B}\right]}^{T}$ is the state vector at time step *k*; $N_{k}^{WB}$ is the matrix associated with the position of the vehicle in the time-discrete form. $N^{Vf}$ is the vehicle system matrix.

Similarly, the EOM of the bridge, Equation (12), is converted into state space form as

(18) $$\Delta X^{B}=N_{c}^{B}\Delta X^{B}+\frac{1}{L^{B}}N_{c}^{M}W_{N}^{B}\left(x\right)\Delta \tilde{\lambda }$$

where $\Delta X^{B}={\left[\begin{array}{cc} \Delta u^{B} & \Delta {\dot{u}}^{B} \end{array}\right]}^{T}$ is the state vector; $N_{c}^{B}$ and $N_{c}^{M}$ are bridge system matrices in time-continuous form as

(19) $$N_{c}^{B}=\left[\begin{array}{cc} 0 & I\\ -{\left(M^{B}\right)}^{-1}K^{B} & -{\left(M^{B}\right)}^{-1}C^{B} \end{array}\right],N_{c}^{M}=\left[\begin{array}{c} 0\\ -{\left(M^{B}\right)}^{-1} \end{array}\right]$$

respectively, where **0** and **I** are zero matrix and identity matrix respectively. Then, the state space EOM of the bridge (Equation (18)) takes a time-discrete form, by multiplying each side of Equation (18) with $\exp(-N_{c}^{B}t)$ [21]. After integration, the converted equation can be given as

(20) $$\Delta X_{k+1}^{B}=N^{B}\Delta X_{k}^{B}+\frac{1}{L^{B}}N^{M}W_{N,k}^{B}\Delta {\tilde{\lambda }}_{k}$$

where *k* in the subscript indicates the time step, $N^{B}$ and $N^{M}$ are the system matrices of the bridge in time-discrete form as

(21) $$N^{B}=\exp\left(-N_{c}^{B}\Delta t\right),N^{M}={\left(N_{c}^{B}\right)}^{-1}\left(N^{B}-I\right)N_{c}^{M}$$

Finally, Equations (16) and (20) are the suitable form for ST-SSI, where matrixes **A** and **C** in Equations (3) and (4) are **A** = $N^{B}$ and **C** = $N^{Vf}N_{k}^{WB}$, respectively.

### 2.5. Identification of Bridge Multi-Order Frequencies

Furthermore, dimensionless velocities are used for evaluating the dynamic properties of a bridge. The ST-SSI method can be applied to the output signal $\Delta P_{k}$ of the system when the dimensionless speed is low, $S_{v1}=\pi v/\omega_{1}^{B}L^{B}<0.1$, where $\omega_{1}^{B}=2\pi f_{1}^{B}$ represents the fundamental frequency of the bridge.

**Figure 2:**
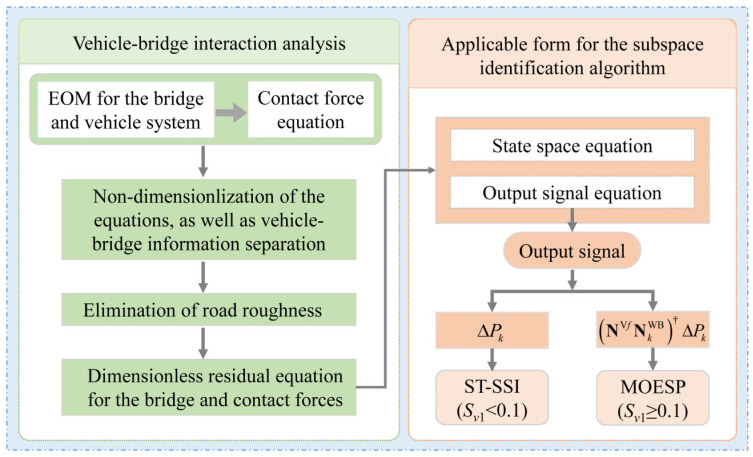
The flowchart of the proposed subspace identification methods.

If the dimensionless vehicle speed exceeds this value, $S_{v1}\geq 0.1$, the dynamic properties of the bridge cannot be ignored. By substituting $W_{N,k}^{B}$ in Equation (20) with $\Phi_{k}$ and setting $N^{Vf}N_{k}^{WB}$ as part of the output signal to solve the time-varying problem, then a suitable form for the MOESP method can be written as

(22) $$\Delta X_{k+1}^{B}=N^{B}\Delta X_{k}^{B}+\frac{1}{L^{B}}N^{M}\Phi_{k}\Delta {\tilde{\lambda }}_{k}$$

(23) $${\left(N^{Vf}N_{k}^{WB}\right)}^{+}\Delta P_{k}=E\Delta X_{k}^{B}$$

where matrixes **A** and **B** in Equation (1) and **C** and **D** in Equation (2) are **A** = $N^{B}$, **B** = $N^{M}/L^{B}$, **C** = **E**, and **D** = **0** respectively; ${\left(\right)}^{†}$ denotes the pseudo-inverse algorithm [43]; **E** is approximately the time-invariant system matrix $E=diag(1,0\ldots ,0)\in R^{2N\times 2N}$; *N* represents the DOFs of the bridge; and $\Phi_{k}$ consists of the estimated shape functions of the bridge (e.g., sinusoidal functions or linear functions). Both the input signal $\Phi_{k}\Delta {\tilde{\lambda }}_{k}$ and the output signal ${\left(N^{Vf}N_{k}^{WB}\right)}^{+}\Delta P_{k}$ are required for the MOESP [43] in identifying bridge frequencies. It should be noted that only the dynamic properties of the bridge are included in the system matrix $N_{c}^{B}$. As a result, the identified frequencies from the system matrix $N_{c}^{B}$ are not affected by the interaction between the vehicle and the bridge and, consequently, are not affected by the time-varying nature of the VBI system. Figure 2 displays the flowchart of the proposed subspace identification methods.

## 3. Numerical Examples

The study performed three numerical examples to test and verify the proposed subspace identification methods considering the dimensionless response of a two-axle *four-degree-of-freedom* vehicle. At the outset, a simply supported bridge is adopted for the validation of the effectiveness and feasibility of ST-SSI and the MOESP in extracting the basic frequency of the bridge. Subsequently, the study establishes two bridges with complex forms, with random vehicle loads being considered, to attempt the extractability of the first two orders of frequencies of large and complex bridges. In order to measure the dimensionless vehicle response, this paper calculates the vehicle response by solving the vehicle–bridge interaction kinematic equation (Equations (6) and (7)) before indirectly identifying the bridge, with four classes of road roughness considered: RRC = 1 (very good), 2 (good), 3 (average), and 4 (poor) [43]. Instead of using records, the road roughness profile herein is generated by a numerical spectrum representation method [45]. As a theoretical verification, the curves of road roughness of different conditions in the calculation model differ only in magnitude yet contain the same components in the frequency domain. For the purpose of stable calculation results, the measured vehicle needs to drive some distance on the uneven road surface before entering the bridge. In addition, the infinite period component in the input and output signals is filtered by removing the mean value. In the end, according to the dimensionless vehicle speed, the ST-SSI (*S_v_*_1_ < 0.1) or MOESP (*S_v_*_1_ ≥ 0.1) method is used to identify the bridge frequency.

### 3.1. Frequency Identification for a Simply Supported Bridge

**Figure 3:**
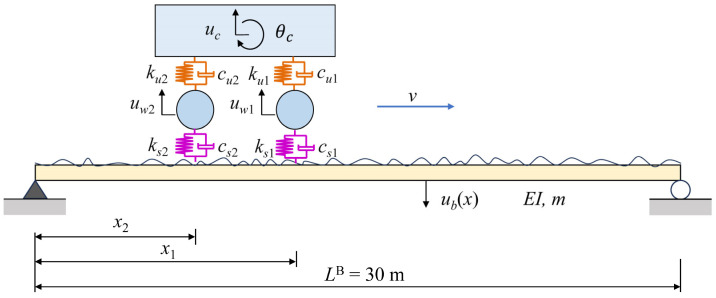
Vehicle-bridge interaction model.

In this section, the feasibility and accuracy of the proposed subspace identification method are verified by numerical tests for a simply supported bridge. The numerically calculated vehicle–bridge interaction model is shown in Figure 3. Table 1 and Table 2 are the parameters and dynamic characteristics of the test vehicle and bridge, respectively. At the same time, in order to reveal the advantages of the proposed subspace identification method compared with conventional SSI in driving speeds, the experiment is set up with four working conditions of vehicle speed, *v* = 10 m/s (36 km/h), 20 m/s (72 km/h), 30 m/s (108 km/h), and 40 m/s (144 km/h), and the corresponding dimensionless speed parameters are *S_v_*_1_ = 0.042, 0.085, 0.127 and 0.170, respectively.

The identification results can be shown by the stabilization diagram [42,43], in which the theoretical bridge frequencies are plotted in dashed lines. Moreover, the hollow circles represent the identified results *f_No_* of the subspace identification method, and the solid ones represent stable identified frequencies *f_n_^id^* [42,43] under a quantitative criterion *ε_f_* = |*f_No_* − *f_No_*_−1_|/*f_No_* < 1%, where *f_No_* represents the identified frequency when the system order is *N_o_*.

Regarding the identification accuracy, the overall results can be assumed to be successful only if the error *ε_n_* = |*f_n_^id^* − *f_n_*^B^|/*f_n_*^B^ is smaller than 5% between the stable identified frequencies and theoretical ones.

**Figure 4:**
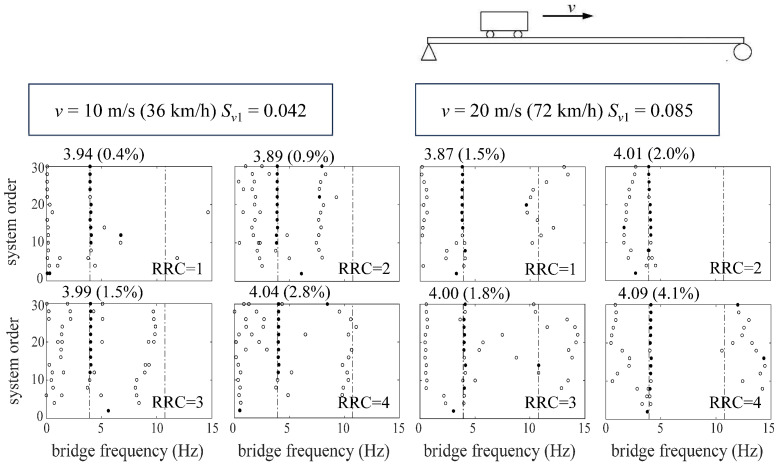
Stabilization diagrams of ST-SSI for the simply supported bridge.

**Figure 5:**
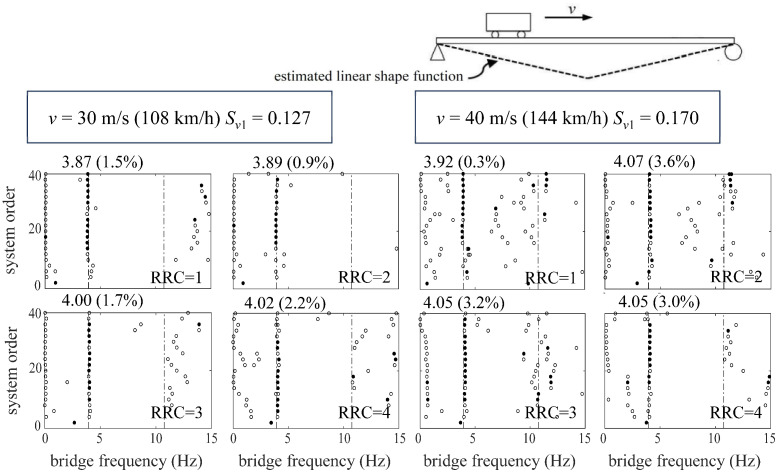
Stabilization diagrams of MOESP for the simply supported bridge.

Figure 4 shows the stabilization diagram of the target bridge extracted by the ST-SSI method from the response of a moving vehicle with normal travelling speeds (*v* = 10 m/s, 20 m/s). Figure 5 shows the results identified by the MOESP method considering higher vehicle speeds (*v* = 30 m/s, 40 m/s). From the above results, it is clear that both the ST-SSI and MOESP methods are able to identify the fundamental frequency of the bridge. Meanwhile, the recognition results under all driving speeds are within the error range. Therefore, the magnitude of the vehicle speed has little effect on the application of both methods under their respective applicable conditions. In addition, the recognition results of the two methods under four road roughness classes also exhibit an excellent effect of filtering road surface noise in the process of identification.

It is worth noting that the second-order frequency of the bridge could not be recognized by either method, a situation that may be due to the simple structural form of the simply supported bridge, whose vibration is mainly controlled by the first-order components. Another possibility is that the loading excitation provided by a vehicle passing over the bridge is small and insufficient to excite effectively the higher-order vibrations of the bridge. In view of this, the effect of the bridge’s structural form and the number of moving vehicles on the identification of the higher-order frequencies remains to be further explored.

### 3.2. Frequency Identification for a Suspension Bridge

**Figure 6:**
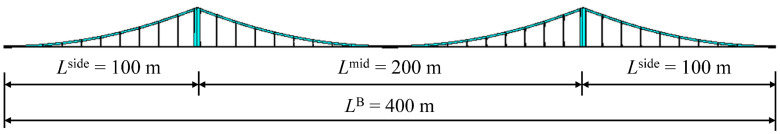
Suspension bridge model.

**Figure 7:**
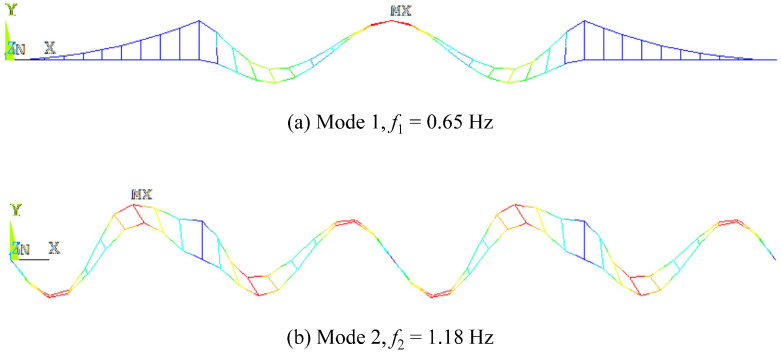
Results of first- and second-order modal analysis of the suspension bridge.

As a case in point to delve into the subspace identification method for large and complex bridges, this section validates the subspace identification for a suspension bridge using the realistic two-axle MDOF vehicle model as the traffic load, with the vehicle properties shown in Table 1 (Section 3.1). The finite element model of the suspension bridge is constructed with the aid of ANSYS, as is shown in Figure 6. Figure 7 displays the results of the first- and second-order modal analysis of the bridge, with its first and second order frequencies being 0.65 Hz and 1.18 Hz, respectively. Table 3 (Section 3.2) shows the properties of the target suspension bridge. The study conducts a numerical calculation of the VBI analysis to replace the actual measurement to acquire the vehicle response as the input signal for the subspace methods for subsequent identification.

#### 3.2.1. Bridge Identification via a Single Vehicle

The study opts for the vehicle speeds of 6 m/s, 12 m/s, and 24 m/s, with its dimensionless counterpart *S_v_*_1_ being 0.062, 0.123, and 0.246, respectively, for the side span and 0.031, 0.062, and 0.123 for the middle span. For such cases, according to different vehicle speeds corresponding to different dimensionless ones, the ST-SSI or the MOESP method is selected for identification.

**Figure 8:**
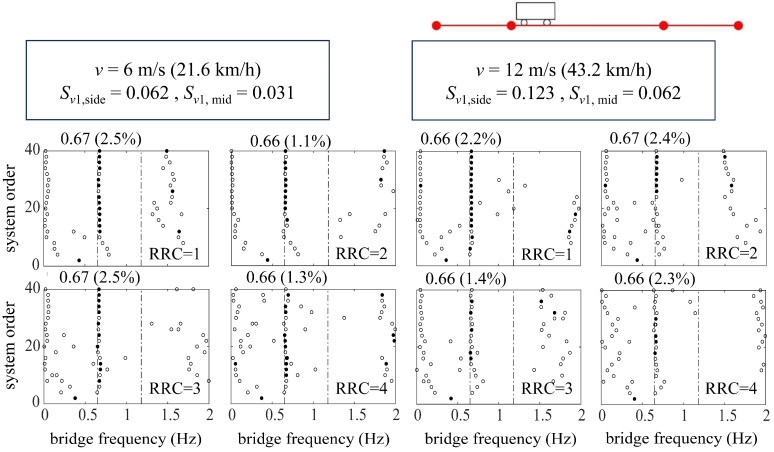
Stabilization diagrams of ST-SSI for the suspension bridge.

**Figure 9:**
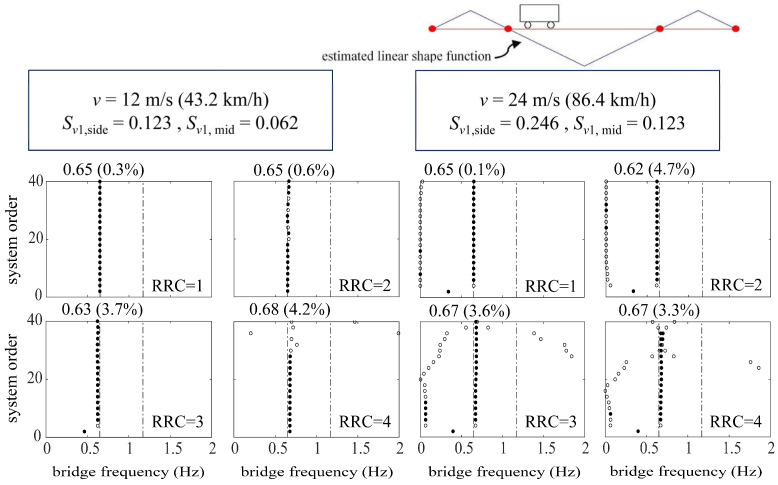
Stabilization diagrams of MOESP for the suspension bridge.

Figure 8 shows the bridge frequencies extracted from a single vehicle traversing at relatively low speeds (*v* = 6 m/s, 12 m/s) over the suspension bridge utilizing the ST-SSI. In the meanwhile, Figure 9 represents the identified results by the MOESP at higher driving speeds (*v* = 12 m/s, 24 m/s). The subspace identification, including both ST-SSI and the MOESP, succeeds in all cases for the fundamental frequency estimation of the bridge, even considering high levels of RRCs. For ST-SSI, the identification effect of the first frequency is clearer and more stable with lower driving speeds and weakens with the deterioration of pavement conditions. It is worth noting that the stability of the MOESP results at the speed of 12 m/s is better than that of the ST-SSI, a situation that could potentially be explained by the value of the dimensionless speed, which is larger than 0.1 for the side span at this point. When the dimensionless speed of the vehicle exceeds this value, the time-varying characteristic of the system does not satisfy the “static” premise assumption of the ST-SSI. On the contrary, the time variation caused by the increase in vehicle speed fits with the assumption of the MOESP, so it is applicable to this case. In addition, it should be noted that there is no indication of any signs of higher-order frequency identification for either the ST-SSI or the MOESP methods. Hence, the excitation from only a single vehicle to the bridge is likely insufficient to activate the higher-order vibration of the bridge. To get this point right, more traffic loads are needed to yield more significant excitations against the bridge.

#### 3.2.2. Bridge Identification Considering Random Traffic

**Figure 10:**
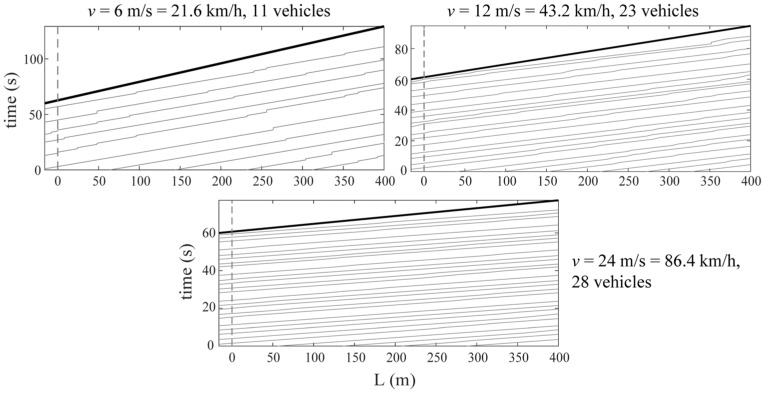
Paths of random traffic for the suspension bridge.

In order to better stimulate the higher-order vibration modes of the bridge, the study applies two subspace identification methods to a more realistic scenario considering the surrounding random traffic flow. Therefore, a Cellular Automation (CA) model is taken to yield the random traffic flow of the bridge, with a moderate flow of service level based on the range of traffic occupancy classified in the highway capacity manual considered [46]. Additionally, the study assigns random parameters to these vehicles, considering weights approximately from 1500 kg to 3000 kg, fundamental frequencies from 1.5 Hz to 3.5 Hz, and a similar damping ratio to the last (measured) vehicle. Figure 10 shows the random traffic paths at three different speeds.

**Figure 11:**
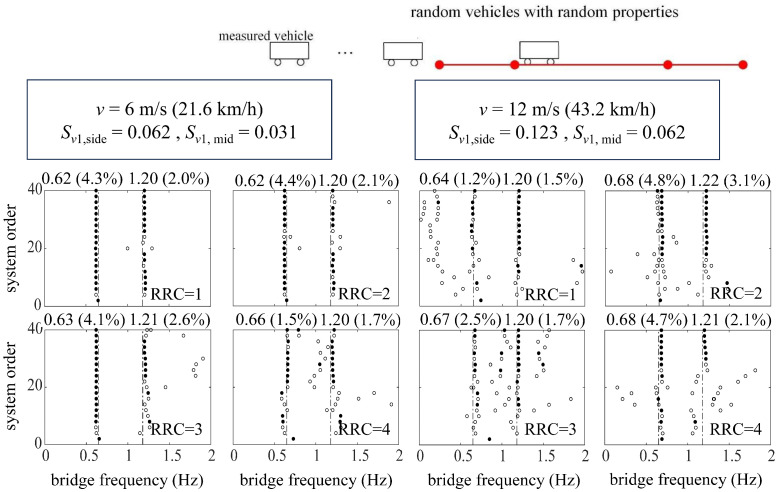
Stabilization diagrams of ST-SSI for the suspension bridge considering random traffic.

**Figure 12:**
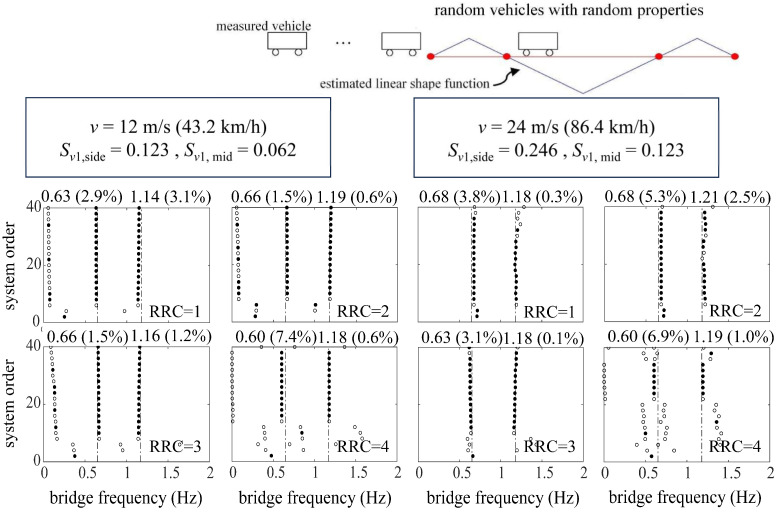
Stabilization diagrams of MOESP for the suspension bridge considering random traffic.

Figure 11 and Figure 12 represent the identification results using ST-SSI and the MOESP considering random traffic, respectively. The ST-SSI method succeeds in the identification of the first two bridge frequencies for all cases when the dimensionless vehicle speeds are small (*S_v_*_1_ < 0.1). On the other hand, for higher speed situations (*S_v_*_1_ ≥ 0.1), most of the first two frequencies can be identified effectively by the MOESP. It should be noted that, for the MOESP, the first-order identified frequency error is relatively obvious when the roughness of the road surface is particularly poor (RRC = 4). Compared with the identification effect via only one vehicle previously, the results indicate that sufficient excitation, including random vibration, is of great significance for bridge modal identification, especially for higher-order modal identification. Multi-point excitations from random traffic offer effective means for activating multi-order modal vibrations of the bridge for identification. It can be concluded that considering that continuous vehicles can better amplify the vibration of the bridge and stimulate the higher-order frequency vibration of the bridge to a certain extent, it is more beneficial for the indirect identification of the bridge frequency. Furthermore, the identification effect of the MOESP still outperforms that of ST-SSI at the speed of 12 m/s, with the clearer and more stable stabilization diagrams displayed by Figure 12. These results are consistent with those obtained by using a single vehicle, showing that the impact of time variation on ST-SSI is gradually marked at this point.

### 3.3. Frequency Identification for a Cable-Stayed Bridge

**Figure 13:**
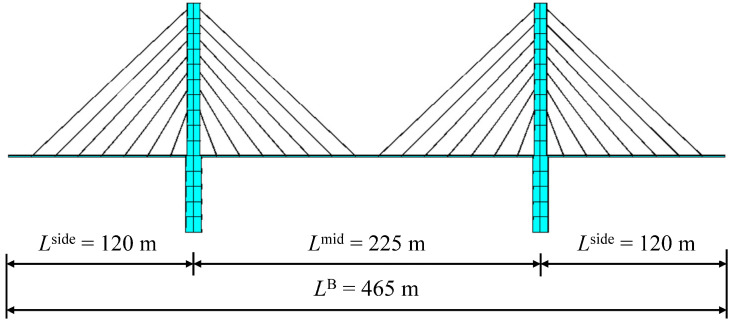
The considered cable-stayed bridge model.

**Figure 14:**
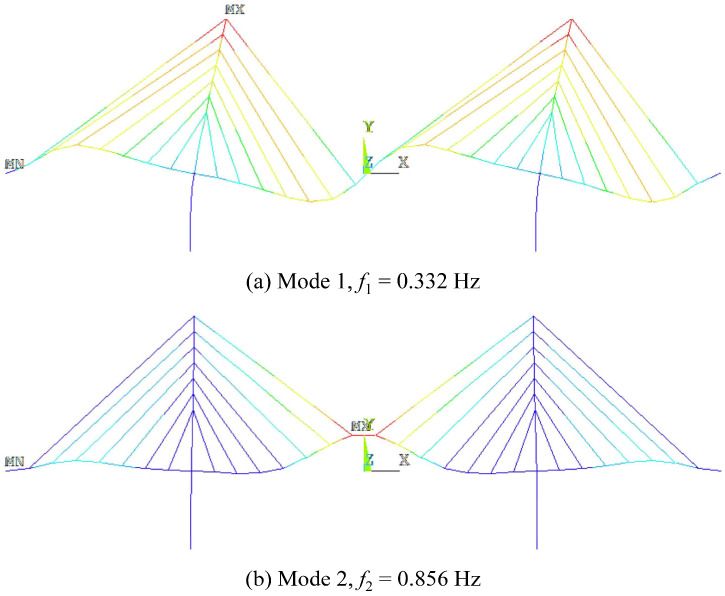
Results of first- and second-order modal analysis of the cable-stayed bridge.

**Figure 15:**
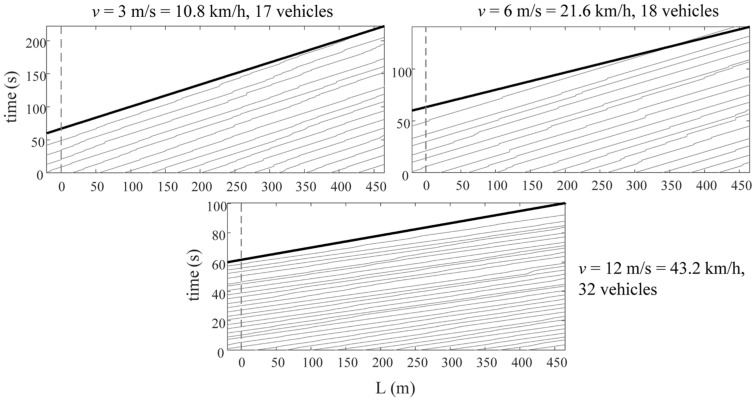
Paths of random traffic for the cable-stayed bridge.

To further verify and enforce the above conclusions, this section discusses the application of the subspace identification to a long-span cable-stayed bridge considering random traffic. The bridge is also established by ANSYS, as is shown in Figure 13. The results of the first- and second-order modal analysis of the cable-stayed bridge are shown in Figure 14. Table 4 represents the characteristics of the bridge. Firstly, the VBI kinematic equation is used to calculate and solve the vehicle response. At the same time, considering three cases of vehicle speed, *v* = 3 m/s, 6 m/s, and 12 m/s, the corresponding dimensionless velocity parameters are as follows: side span *S_v_*_1,side_ = 0.050, 0.100, and 0.200 and middle span *S_v_*_1,mid_ = 0.027, 0.054, and 0.107. In this experiment, the parameters of the measured vehicle are shown in Table 1. Similar to Section 3.2.2, the random vehicles simulate a normal traffic flow under the bridge service state, and the random vehicle parameters were also randomly assigned, with the vehicle weight being random, from 1500 kg to 3000 kg, the first-order frequency being random, from 1.5 Hz to 3.5 Hz, and the damping ratio being similar to that of the last (measured) vehicle. Before the measured vehicle enters the bridge, a one-minute random vehicle simulation is carried out. Figure 15 shows the driving paths of random vehicles at different vehicle speeds, where the thick black line represents the measured vehicle.

**Figure 16:**
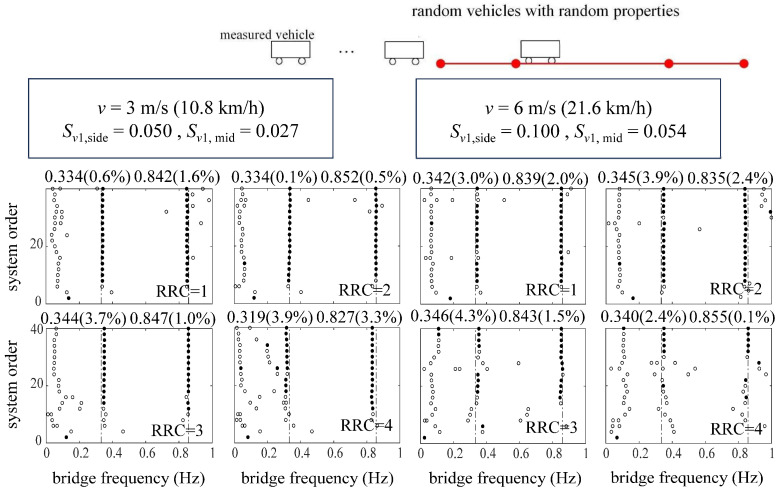
Stabilization diagrams of ST-SSI for a cable-stayed bridge considering random traffic.

**Figure 17:**
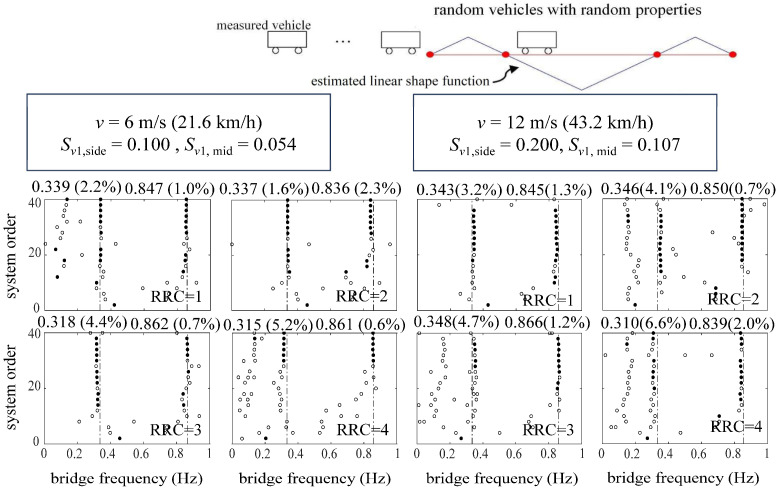
Stabilization diagrams of MOESP for a cable-stayed bridge considering random traffic.

Figure 16 and Figure 17 show the identification results of the ST-SSI and the MOESP methods, respectively. It can be seen from Figure 16 that the ST-SSI method can successfully identify the first two frequencies of the bridge under all working conditions, including considering high road roughness and random vehicles. With the vibration of the bridge amplified by the random traffic flow, the MOESP method can identify the basic frequency of the bridge, and it exhibits a good identification effect on the second-order frequency of the bridge. In the case of a combination of high speed (*v* = 12 m/s) and poor road roughness (RRC = 4), the MOESP sees a relatively conspicuous identified error in the identification of the first-order frequency of the bridge. However, to sum up, a random traffic flow remains beneficial to the identification of higher-order frequencies.

In conclusion, the successful identification of the cable-stayed bridge frequencies by using the subspace identification methods considering the dimensionless response of two-axle vehicles is of great significance for the wide application of this method to large-scale and rapid bridge screening by ordinary vehicles with normal driving speeds in practical scenarios.

## 4. Conclusions

This study theoretically derives subspace identification methods of using a two-axle vehicle to identify bridge frequencies. This study focuses on developing formulations for subspace identification techniques, i.e., improved ST-SSI and MOESP, to the VBI problem involving a single two-axle vehicle and a series of random vehicle traffic flows. No information from the bridge is required for the proposed subspace identification techniques, since they rely solely on the vehicle’s information. The proposed method investigates and finally excludes the adverse factors for indirect identification of bridge frequency in the process of theoretical model derivation. Primarily, it eliminates the adverse effect due to RRCs by processing the response from two wheels of one traversing vehicle, instead of using vehicle responses from two traverses considering similar speeds and following same driving path. Compared with the previous ST-SSI and MOESP, the procedure for data processing completely removes the negative noise for the identification process, so the scheme does not rely on vehicle properties. More importantly, the introduction of a dimensionless description of the VBI system’s response leads to an enhanced robustness of the vehicle driving speeds that is manifested by the fact that the ST-SSI method can be applied to the vehicle traveling at normal driving speeds (*S_v_*_1_ < 0.1), while the MOESP method has a wider redundancy to vehicle speeds, and thus, can be applied to detection for highway bridges (*S_v_*_1_ ≥ 0.1). To this end, the proposed method can be applied to different vehicle types or properties, to different vehicle numbers or traffic flow distributions, as well as to varied vehicle travelling speeds.

The numerical examples examined three bridges with different levels of DOFs and scales, a simple bridge, suspension bridge, and cable-stayed bridge. The proposed subspace identification method can accurately and effectively extract the dominated fundamental frequency of the simply supported bridge, provided that the response of a single moving vehicle crossing the bridge with a single passage is known. For more complicated bridge structures with denser modes, the presence of random traffic can stimulate the bridge to produce a larger amplitude of vibration. The more vehicles passing over, the stronger the excitation effect on the bridge. Hence, the consideration of random vehicle flows is conducive to the multi-order frequency identification of complicated bridges. The successful identification of the first few order frequencies of the above bridges using the subspace identification technique is of great significance for the application of vehicle scanning technology in practice that it is not confined in terms of vehicle speed, vehicle parameters, road surface conditions, surrounding traffic, or bridge types.

## Figures and Tables

**Table 1:** Properties of the measured two-axle MDOF vehicle [42].

Vehicle Parameter	Symbol	Value	Unit
Mass of car body	m_c_	4480	kg
Moment of inertia of car body	I_c_	5516	kg·m^2^
Mass of front wheel	m_w1_	800	kg
Mass of rear wheel	m_w2_	710	kg
Upper vertical spring stiffness	k_u1_/k_u2_	399,000	N/m
Lower vertical spring stiffness	k_s1_/k_s2_	351,000	N/m
Upper vertical damping coefficient	c_u1_/c_u2_	23,210	N·s/m
Lower vertical damping coefficient	c_s1_/c_s2_	800	N·s/m
Front wheel-center distance	*L*_1_	2.6	m
Rear wheel-center distance	*L*_2_	3.0	m
First frequency		1.95	Hz
Second frequency		3.82	Hz

**Table 2:** Properties of the simply supported bridge.

Length *L*^B^ (m)	Flexural Stiffness *EI* (kN·m^2^)	Per-Unit-Mass *μ* (kg/m)	$f_{1}^{B}$	$f_{2}^{B}$
30	1.80 × 10^11^	1.50 × 10^3^	3.93	10.75

**Table 3:** Properties of the suspension bridge.

Length *L*^B^ (m)	Flexural Rigidity *EI* (kN·m^2^)	Per-Unit-Mass *μ* (kg/m)	$f_{1}^{B}$	$f_{2}^{B}$
400	9.91 × 10^11^	2.14 × 10^3^	0.65	1.18

**Table 4:** Properties of the cable-stayed bridge.

Length *L*^B^ (m)	Flexural Rigidity *EI* (kN·m^2^)	Per-Unit Mass *μ* (kg/m)	$f_{1}^{B}$	$f_{2}^{B}$
465	2.09 × 10^13^	2.15 × 10^5^	0.332	0.856

## Data Availability

Data are contained within the article.
